# Zika virus recruits karyopherin α6 for efficient replication via NS2B

**DOI:** 10.1128/jvi.02009-25

**Published:** 2026-05-20

**Authors:** Peixi Chang, Jia He, Bhargava Teja Sallapalli, Liping Yang, Yanjin Zhang

**Affiliations:** 1 Molecular Virology Laboratory, Virginia-Maryland College of Veterinary Medicine, University of Maryland 1068 , College Park, Maryland, USA; 2 Department of Molecular Medicine and Therapeutics, The Ohio State University College of Medicine 2647 , Columbus, Ohio, USA; Wake Forest University School of Medicine, Winston-Salem, North Carolina, USA

**Keywords:** Zika virus, karyopherin, KPNA6, relocation, NS2B, viral replication, proviral factor, virus-host interaction

## Abstract

Zika virus (ZIKV) is a mosquito-borne flavivirus that caused an epidemic in the Americas in 2015–2016, accompanied by severe neurological manifestations, including congenital Zika syndrome and Guillain-Barré syndrome. Our previous data demonstrate that the protein level of karyopherin α6 (KPNA6), a transport factor in nucleocytoplasmic trafficking, increases in ZIKV-infected cells, and that KPNA6 depletion reduces ZIKV replication. Here, we found that ZIKV infection led to the relocation of KPNA6 to the perinuclear region. KPNA6 was observed to partially co-localize with double-stranded RNA, an intermediate in the replication of positive-sense, single-stranded RNA viruses. Further studies revealed that the ZIKV protein NS2B mediates the relocation of KPNA6 and that both proteins interact, as indicated by co-immunoprecipitation. The mutagenesis studies showed that KPNA6 co-precipitated NS2B via its major groove, and that, in turn, NS2B pulled down KPNA6 via its C-terminus. Furthermore, two residues, P115 and G119, in NS2B were identified as critical for interaction with KPNA6, and mutations in either residue abolished virus replication. These results demonstrate that ZIKV induces the relocation of KPNA6 via NS2B, uncovering a novel ZIKV-host interaction to recruit a host proviral factor to facilitate viral proliferation.

**IMPORTANCE:**

Like most other positive-sense RNA viruses, Zika virus (ZIKV) replicates in the cytoplasm; however, the mechanisms by which it recruits host factors for efficient proliferation remain elusive. Our results demonstrate that ZIKV induces the relocation of karyopherin α6 (KPNA6) to the perinuclear region, likely mediated by the viral protein NS2B. Further analysis shows that KPNA6 interacts with NS2B, with two critical residues in NS2B required for the interaction. A mutation in these two residues abolishes virus replication. Despite the absence of a predicted nuclear localization signal sequence in NS2B, this protein was found to bind the major groove of KPNA6. These findings shed light on the interaction between ZIKV and a proviral host factor, providing valuable insights that may inform the development of future antiviral strategies.

## INTRODUCTION

Zika virus (ZIKV) is a member of the *Flavivirus* genus in the family *Flaviviridae*, which includes several other global human pathogens, such as dengue virus, yellow fever virus, and West Nile virus (1, 2). ZIKV is a mosquito-borne virus that caused an epidemic in the Americas during 2015–2016, leading to severe neurological manifestations, such as congenital Zika syndrome (CZS) and Guillain-Barré syndrome (3–5). CZS is characterized by microcephaly, subcortical calcifications, ocular abnormalities, arthrogryposis, cognitive deficits, and other developmental defects in newborns (5). ZIKV has become endemic and co-circulates with the dengue virus in tropical regions (6, 7), with a recent outbreak in India (8). Accordingly, ZIKV infection is a public health concern, and its associated neurological illnesses pose an important healthcare issue that requires attention. However, there is currently no effective treatment or vaccine commercially available to control and prevent ZIKV infection (9).

ZIKV is an enveloped positive-sense, single-stranded RNA [(+)ssRNA] virus with a genome of 10.8 kb. Upon entry, the viral RNA genome serves as mRNA to translate into a polyprotein, which is cleaved into three structural proteins: capsid (C), precursor membrane (prM), and envelope (E), and seven non-structural proteins: NS1, NS2A, NS2B, NS3, NS4A, NS4B, and NS5 (10). An intermediate double-stranded RNA (dsRNA) is generated during the viral genome replication. RNA synthesis occurs in a virus-induced membranous structure derived from the endoplasmic reticulum (ER) (11). Since the membranous structure is formed in the cytoplasmic space, little is known about whether ZIKV RNA synthesis needs the nucleocytoplasmic trafficking system. Our earlier data demonstrate that the protein level of karyopherin α6 (KPNA6), a transport factor in nucleocytoplasmic trafficking (12), increases in ZIKV-infected cells, and that KPNA6 depletion reduces ZIKV replication (13).

KPNA6 is a member of the karyopherin protein family, which mediates nucleocytoplasmic trafficking and is critical for the movement and subcellular localization of macromolecules within cells (12, 14). Karyopherins facilitate the nuclear import and export of numerous proteins, including transcription factors involved in cell growth, host immunity, and defense. In the classical nuclear import pathway, a cytoplasmic cargo bearing a nuclear localization signal (NLS) is first recognized by an adaptor protein, karyopherin α, which then associates with karyopherin β1 (KPNB1) (12, 14). Then, the ternary import complex of karyopherin β/α/cargo translocates through the nuclear pore complex into the nucleus.

Human karyopherin α (KPNA) comprises seven isoforms grouped into three subfamilies (12). KPNA1, 5, and 6 belong to the same subfamily and share 74% amino acid identity. All KPNA isoforms have similar structures, featuring a flexible N-terminal importin β-binding (IBB) domain, followed by 10 highly structured tandem armadillo (ARM) repeats, and a C-terminal domain. The helical ARM repeats form a major groove and a minor groove for cargo NLS binding (12, 15). Despite their structural similarities, each KPNA isoform has its own cargo preference. KPNA6 is known to transport multiple proteins to the nucleus (16, 17), including the signal transducer and activator of transcription protein 3 (STAT3) (18) and Keap1, a cytosolic inhibitor of the transcription factor Nrf2 during the antioxidant response (19). In addition, KPNA6 is essential for zygotic genome activation and early mouse development, as embryos lacking KPNA6 arrest at the two-cell stage (20).

We previously discovered that KPNA6 is crucial for the efficient replication of ZIKV and porcine reproductive and respiratory syndrome virus (PRRSV) (13). Here, we demonstrate that ZIKV induces the relocation of KPNA6 to the perinuclear region, partially co-localizing with dsRNA, and that ZIKV protein NS2B can mediate this relocation. Further studies revealed the parts of NS2B and KPNA6 involved in their interaction and identified two critical residues in NS2B required for the association. Notably, mutations in either of the two residues abolished virus replication. These results uncover a novel ZIKV-host interaction recruiting a host proviral factor to facilitate efficient viral replication.

## RESULTS

### ZIKV induces the translocation of KPNA6 to the perinuclear region

**Fig 1:**
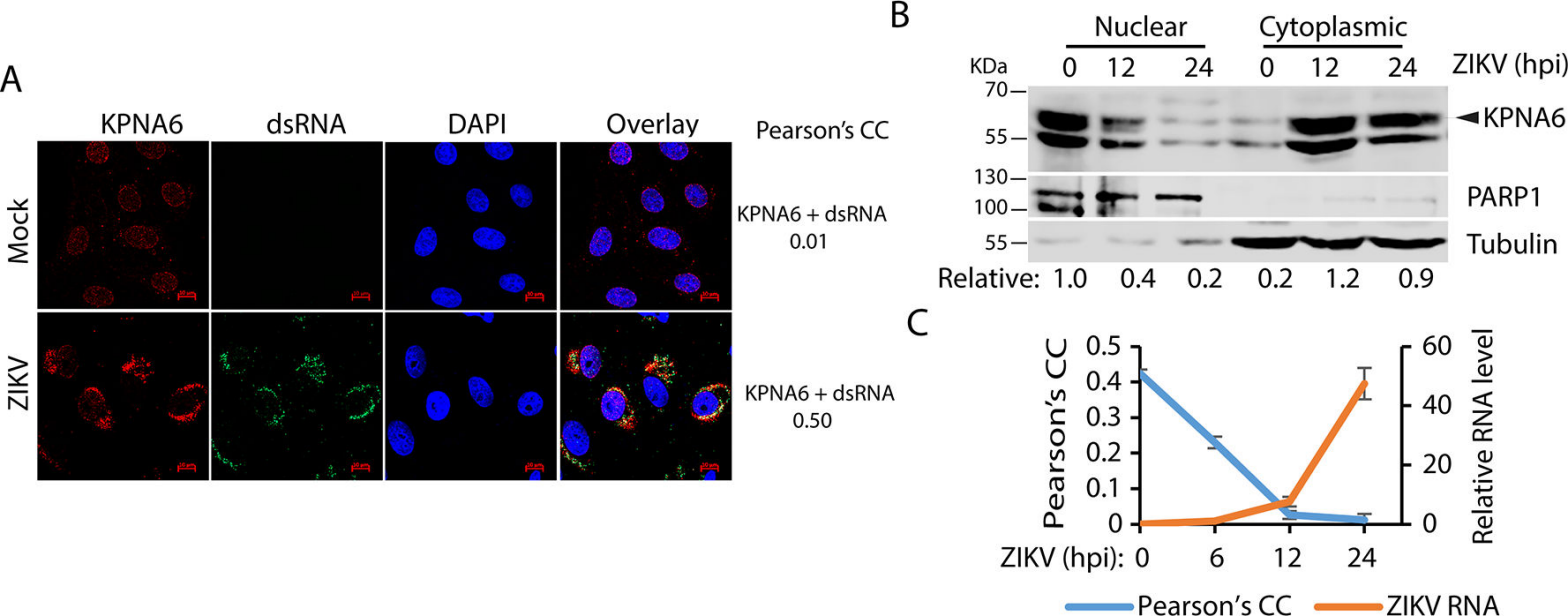
ZIKV induces relocation of KPNA6 to the perinuclear region. (**A**) KPNA6 partially co-localizes with ZIKV dsRNA in Vero cells. The cells were infected with the ZIKV PRVABC59 strain at a multiplicity of infection (MOI) of 10 and fixed for IFA 24 hpi with the antibodies against dsRNA and KPNA6. An overlay of dsRNA, KPNA6, and DAPI nuclear staining is shown. The scale bars in the lower right of each image denote 10 μm. Pearson’s CC of KPNA6 and dsRNA is indicated on the right. (**B**) KPNA6 in the cytoplasmic fraction increases as ZIKV infection progresses. Nuclear and cytoplasmic fractionation was confirmed using antibodies against PARP1 and tubulin, respectively. Relative KPNA6 levels are shown below the images, normalized to PARP1 and tubulin for the nuclear and cytoplasmic fractions, respectively. Molecular weight markers are indicated to the left of the images. (**C**) Pearson’s CC of KPNA6 and DAPI decreases as ZIKV infection progresses. Vero cells were infected with ZIKV at an MOI of 10 and fixed for IFA at 0, 6, 12, and 24 hpi. The average Pearson’s CC of KPNA6 and DAPI is shown from 10 representative cells in confocal microscopy images analyzed with the Carl Zeiss Zen program. Error bars denote variation. ZIKV RNA levels in the infected cells were determined by RT-qPCR.

Our earlier work shows that both ZIKV and PRRSV infection lead to the elevation of KPNA6 protein levels and that KPNA6 depletion impairs their replication (13). As a transport factor involved in nucleocytoplasmic trafficking, KPNA6 is typically associated with nuclear transport; however, ZIKV primarily replicates in the cytoplasm. This raised the question of whether the viral infection alters the subcellular localization of KPNA6. To test this speculation, we infected Vero cells with the ZIKV PRVABC59 strain and conducted an immunofluorescence assay (IFA) with an antibody against KPNA6 24 h post-infection (hpi). The IFA result showed a perinuclear pattern of KPNA6 in ZIKV-infected cells, whereas the majority of KPNA6 remains in the nucleus of the mock-infected cells (Fig. 1A). In addition, IFA with an antibody against dsRNA, a replication intermediate generated during RNA synthesis of most (+)ssRNA viruses, indicates that KPNA6 partially co-localizes with the ZIKV dsRNA in the perinuclear region of infected cells, with a Pearson’s correlation coefficient (Pearson’s CC) of 0.5.

To test whether the subcellular localization of KPNA6 changed along with the course of virus infection, we infected Vero cells with the ZIKV PRVABC59 strain and collected cells at 0, 12, and 24 hpi. We then fractionated the nuclear and cytoplasmic fractions and performed Western blotting (WB). Using tubulin as a cytoplasmic control and PARP1 as a nuclear control, the results demonstrated a dynamic shift in KPNA6 localization during ZIKV infection. As the infection progressed, KPNA6 levels decreased in the nucleus and increased in the cytoplasm, whereas the majority of KPNA6 remained in the nucleus at 0 hpi (Fig. 1B). We also conducted IFA on ZIKV-infected Vero cells and analyzed the Pearson’s CC between KPNA6 and DAPI nuclear staining. The results indicate that KPNA6 co-localization with DAPI decreases from 0 to 24 hpi, while ZIKV RNA levels increase over time (Fig. 1C), suggesting that ZIKV infection induces the relocation of KPNA6 from the nucleus to the cytoplasm.

### ZIKV protein NS2B induces relocation of KPNA6 and co-precipitates KPNA6

**Fig 2:**
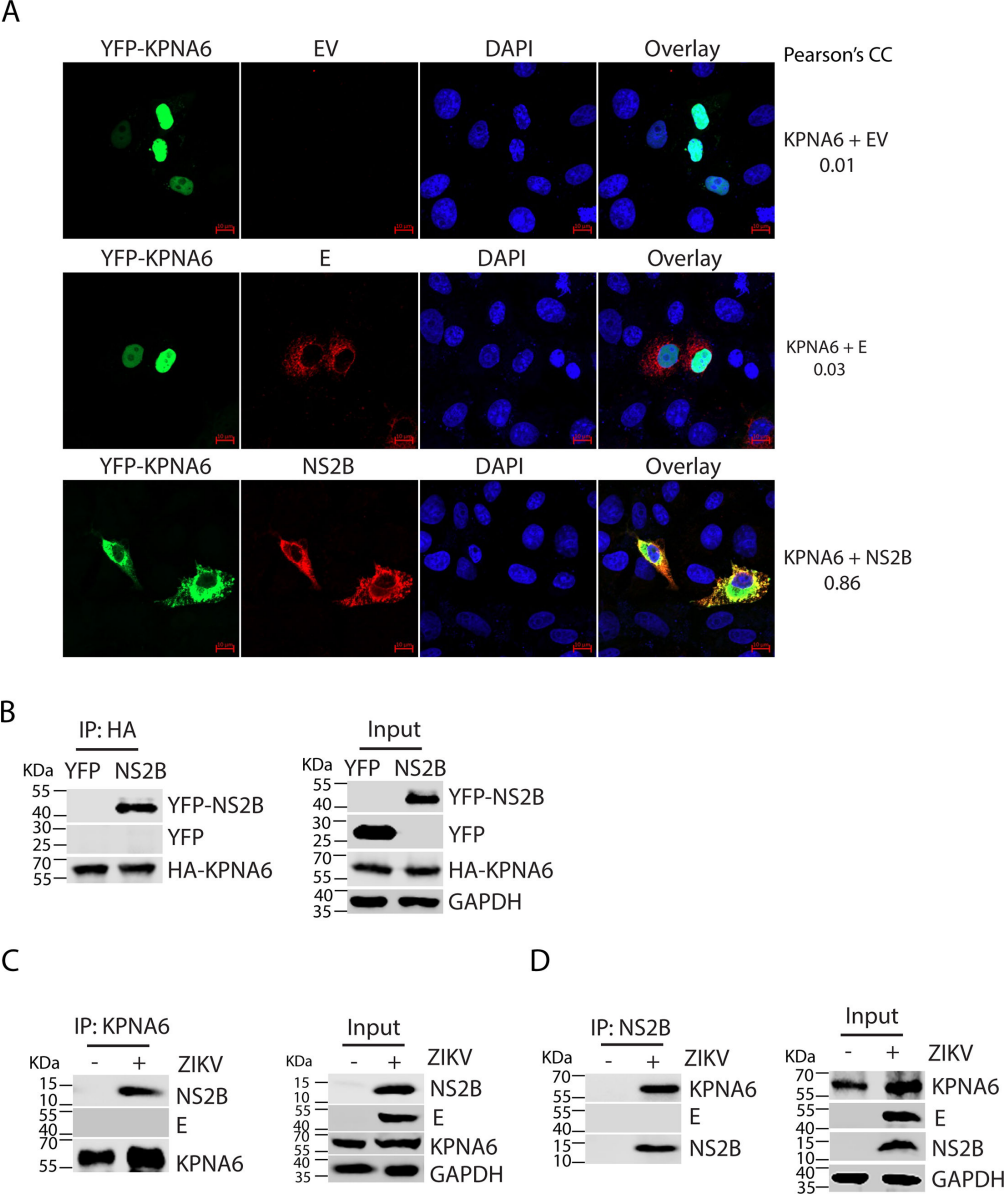
The ZIKV protein NS2B induces the relocation of KPNA6 and interacts with it. (**A**) NS2B induces the relocation of KPNA6 in co-transfected cells. Vero cells were co-transfected with plasmids of YFP-tagged KPNA6 and Myc-tagged NS2B and E or empty vector (EV). The cells were fixed for IFA with an antibody against the Myc tag 24 h post-transfection. The overlays of NS2B or E, KPNA6, and DAPI are shown. The scale bars in the lower right of each image denote 10 μm. Pearson’s CC is shown on the right. (**B**) KPNA6 IP co-precipitates NS2B in transfected HEK293T cells. The cells were co-transfected with hemagglutinin (HA)-tagged KPNA6 and YFP-tagged NS2B, then harvested for IP at 48 hpt. (**C**) KPNA6 IP co-precipitates NS2B from ZIKV-infected Vero cells but not ZIKV E protein. (**D**) KPNA6 is present in the co-IP precipitate of NS2B, whereas ZIKV E is not. The Vero cells were infected with the ZIKV PRVABC59 strain at an MOI of 10 and harvested for IP at 24 hpi.

The results above show that KPNA6 partially co-localizes with dsRNA within replication complexes. To identify the viral protein(s) responsible for relocating KPNA6 to the perinuclear region, we co-transfected Vero cells with yellow fluorescent protein (YFP)-tagged KPNA6 and plasmids encoding individual ZIKV proteins, followed by live-cell fluorescence microscopy. The results showed that NS2A, NS2B, NS4A, and NS4B induced varying levels of YFP-tagged KPNA6 distribution to the perinuclear region (Fig. S1A and B). Among these four proteins, NS2B induced the most prominent KPNA6 relocation. We therefore focused on subsequent analysis of the effect of NS2B on KPNA6. IFA results showed that NS2B strongly co-localized with the YFP-KPNA6 in the perinuclear region in co-transfected Vero cells, with a Pearson’s CC of 0.86, whereas cells expressing the structural protein E still showed predominant nuclear localization of KPNA6 (Fig. 2A). Co-expression of the other individual viral proteins was also confirmed (Fig. S1C). These results indicate that NS2B is potentially the main player responsible for the ZIKV-mediated KPNA6 cytoplasmic relocation.

The result above prompted us to hypothesize that KPNA6 might interact with NS2B. To confirm this hypothesis, we co-transfected HEK293T cells with plasmids encoding HA-tagged KPNA6 and YFP-tagged NS2B. Co-immunoprecipitation (IP) of KPNA6 with HA-tag antibody was done, followed by WB. The result showed that KPNA6 co-precipitated NS2B but not the YFP control (Fig. 2B), indicating an interaction between KPNA6 and NS2B. This interaction was also verified in ZIKV-infected cells, where KPNA6 antibody co-precipitated NS2B but not structural protein E (Fig. 2C). Reciprocally, IP with an NS2B antibody also co-precipitated KPNA6 but not E (Fig. 2D), indicating a specific interaction of KPNA6 with NS2B during ZIKV infection.

### The major NLS-binding motif of KPNA6 interacts with ZIKV NS2B

**Fig 3:**
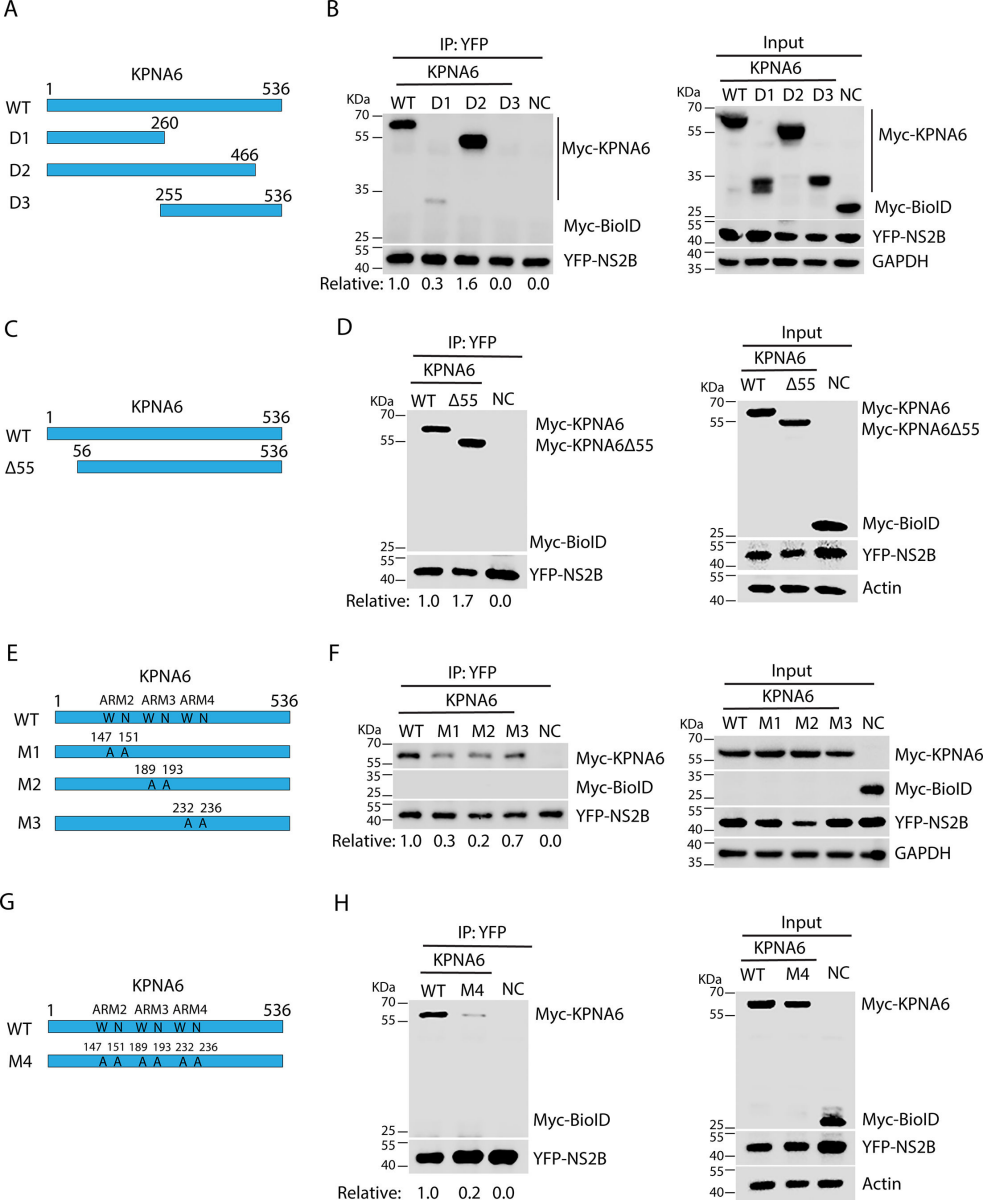
The N-terminal half of KPNA6 interacts with ZIKV NS2B. (**A**) Schematic illustration of KPNA6 truncation constructs. Numbers above the bars denote the positions of amino acid residues of KPNA6. (**B**) KPNA6-D1 and D2 interact with NS2B, while D3 loses the interaction. HEK293T cells were co-transfected with YFP-tagged NS2B and Myc-tagged KPNA6 truncations or with negative control Myc-tagged BioID (NC). The cell lysates were harvested at 48 hpt for co-IP with GFP antibody. The relative co-precipitation efficiency of KPNA6, shown below the IP images, was determined by normalizing the KPNA6 and NS2B levels in the IP to their respective input levels. (**C**) Schematic illustration of KPNA6Δ55 truncation construct. (**D**) KPNA6Δ55 co-precipitates NS2B. (**E**) Schematic illustration of mutant KPNA6 constructs. Residues Trp (W) and Asn (N) for the substitution and corresponding residue Ala (A) in the mutant constructs are indicated. (**F**) Mutant KPNA6-M1 and M2 reduce interaction with NS2B. (**G**) Schematic illustration of the combined mutant KPNA6-M4 construct. (**H**) Mutant KPNA6-M4 reduces interaction with NS2B. HEK293T cells were co-transfected with YFP-tagged NS2B and Myc-tagged KPNA6 and mutants. The cell lysates were harvested at 48 hpt for co-IP with GFP antibody.

NS2B lacks a predicted NLS and is cytoplasmic in ZIKV-infected cells. This prompted us to define the NS2B-binding site in KPNA6, which has 10 ARM repeats forming the major and minor grooves for cargo NLS binding. To determine the binding motif in KPNA6 for the NS2B interaction, we constructed three deletion mutants based on KPNA6 structure (Fig. 3A). HEK293T cells were co-transfected with plasmids encoding YFP-tagged NS2B and the Myc-tagged wild-type or truncated KPNA6, followed by IP with a GFP antibody to precipitate YFP-NS2B. The results showed that KPNA6-D1 and D2, but not D3, were present in the NS2B precipitates (Fig. 3B). However, KPNA6-D2 contains all the residues in D1 and all but the last 70 residues from D3. So, the conclusion was that the N-terminal half of KPNA6 was responsible for the interaction with NS2B. Since the IBB domain is located in the N-terminus of KPNA6, we next tested whether this domain contributes to NS2B binding. A deletion mutant lacking the IBB domain, KPNA6Δ55, was constructed (Fig. 3C). Co-transfection of HEK293T cells with KPNA6Δ55 and NS2B plasmids, followed by IP, was performed. The results showed that, like full-length KPNA6, KPNA6Δ55 interacts with NS2B (Fig. 3D), suggesting that the KPNA6 IBB domain is dispensable for this interaction.

KPNA6 has 10 ARM repeats, each containing three α-helices (H1–H3). The H3 helices line the inner surface of the major and minor grooves for NLS binding, containing several Asn and Trp residues highly conserved across all KPNAs (21). The ARM repeats 2–4 on the N-terminal half form the major groove, and the ARM repeats 6–8 on the C-terminal half form the minor groove. Site-directed mutagenesis of the asparagine (Asn or N) and tryptophan (Trp or W) residues in the ARM repeats 2, 3, and 4 was done to determine whether they are involved in the interaction with NS2B (Fig. 3E). Co-transfection of HEK293T cells with these KPNA6 mutants and YFP-tagged NS2B plasmids was done, followed by IP with GFP antibody. WB analysis of the IP precipitate showed that KPNA6-M1 and M2 had much lower levels than wild-type KPNA6 and KPNA6-M3 (Fig. 3F), suggesting that the Asn and Trp residues in ARM repeats 2 and 3 contribute more to the NS2B interaction than those in ARM repeat 4. To further assess whether the three ARM repeats within the major binding groove contribute cooperatively to the NS2B binding, we constructed a mutant, KPNA6-M4, containing substitutions of the conserved N and W residues in ARM2, ARM3, and ARM4 (Fig. 3G). HEK293T cells were co-transfected with the plasmids encoding the KPNA6-M4 mutant and YFP-tagged NS2B, and then IP was performed with the GFP antibody. The IP result showed that, compared to wild-type KPNA6, mutant KPNA6-M4 was present at a much lower level in the NS2B precipitate (Fig. 3H). These results indicate that the major groove of KPNA6 is potentially responsible for the NS2B binding.

### The C-terminal domain of NS2B is responsible for the interaction with KPNA6

**Fig 4:**
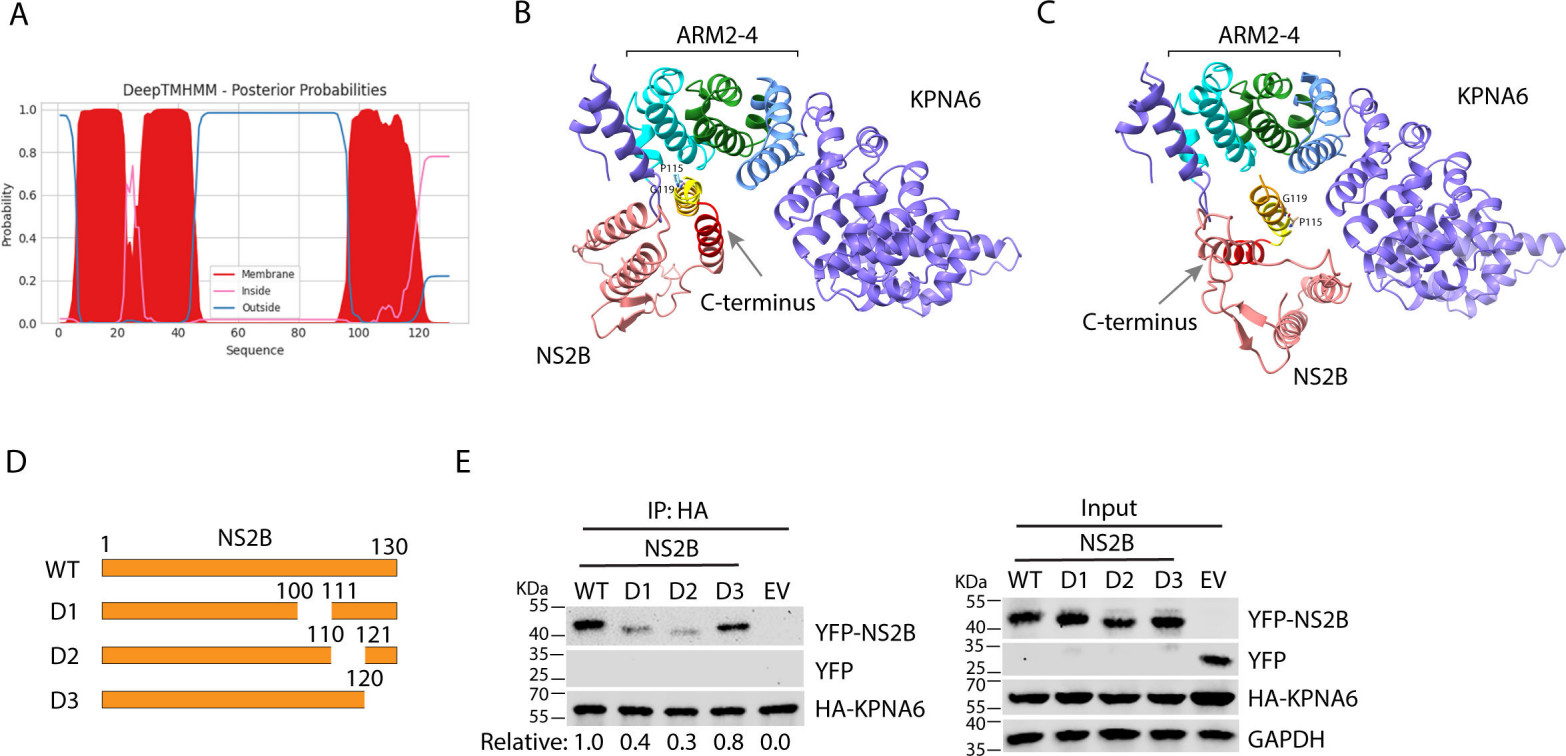
The C-terminal domain of NS2B interacts with KPNA6. (**A**) Transmembrane domain prediction of NS2B by the DeepTMHMM analysis. The number on the *x*-axis indicates the number of amino acid residues of NS2B. (**B and C**) the top view of the interaction model between NS2B and KPNA6 from the ZDOCK server (**B**) and HADDOCK web server (**C**). The pink indicates NS2B, and the purple indicates KPNA6. The major groove ARMs of KPNA6 are highlighted in cyan (ARM2), green (ARM3), and blue (ARM4), respectively. The arrow indicates the C-terminus of NS2B. The C-terminal amino acids of NS2B are highlighted in red (aa 101–110), yellow (aa 111–120), and orange (aa 121–130), respectively. Residues proline 115 (P115) and glycine 119 of NS2B are shown in stick representation. (**D**) Schematic illustration of the constructs of NS2B truncations. The numbers above the lines indicate the position of NS2B amino acid residues. (**E**) Deletions between 100 and 120 of NS2B (D1 and D2) reduce the interaction with KPNA6. HEK293T cells were co-transfected with HA-tagged KPNA6 and YFP-tagged NS2B or deletion constructs, or EV, and the cell lysates were harvested 48 hpt for co-IP with HA-tag antibody. The relative co-precipitation efficiency of NS2B, shown below the IP images, was determined by normalizing the NS2B and KPNA6 levels in the IP to their respective input levels.

The results above indicate that NS2B interacts with KPNA6. We then wondered which part of NS2B mediates its interaction with KPNA6. NS2B is a 14 kDa protein with a hydrophilic central loop (aa 45–95) and transmembrane helices at the protein’s N- and C-termini (22–25). As a cofactor of the NS2B-NS3 protease, NS2B is anchored in the ER, with the hydrophilic loop on the cytoplasmic side, where it interacts with the serine protease domain of NS3 (26, 27). We conducted a secondary structural analysis of ZIKV PR NS2B by DeepTMHMM analysis, showing transmembrane domains in both N- and C-termini (Fig. 4A). A protein-protein interaction model analysis from the ZDOCK server suggests that the C-terminal domain of NS2B might interact with KPNA6 (Fig. 4B), as the C-terminus of NS2B was predicted to participate in binding across all top 10 docking models. To further examine the potential interaction between NS2B and KPNA6, protein–protein docking was performed using the HADDOCK web server. The analysis produced a top-ranked complex that closely resembled the model obtained from the ZDOCK prediction (Fig. 4C). To test this prediction, we generated three deletion mutants of NS2B: D1, D2, and D3, each with a deletion of 10 residues (Fig. 4D). HEK293T cells were co-transfected with constructs of HA-tagged KPNA6 and wild-type or truncated YFP-tagged NS2B, followed by IP using an HA-tag antibody. The IP result showed that deletion mutants NS2B-D1 and D2 in the KPNA6 precipitates were much lower than those of wild-type NS2B and the mutant NS2B-D3 (Fig. 4E), indicating that residues 101–120 of NS2B were possibly responsible for the interaction with KPNA6.

### The amino acid residues P115 and G119 of NS2B are essential for interacting with KPNA6

**Fig 5:**
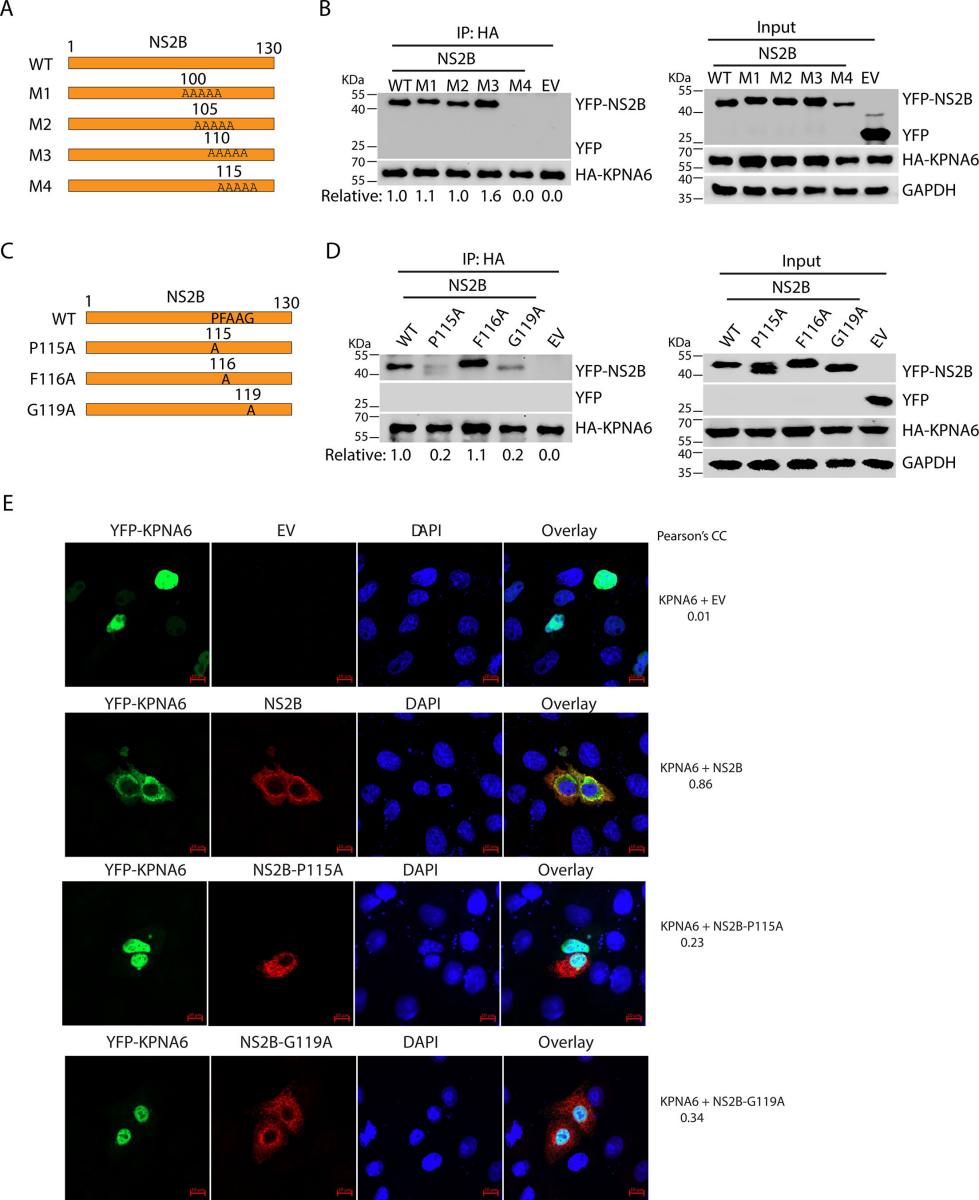
The amino acid residues P115 and G119 of NS2B are essential for NS2B interacting with KPNA6. (**A**) Schematic illustration of alanine scanning mutagenesis between aa 100–119 of NS2B and mutants derived from it. Numbers above the bars denote the positions of amino acid residues of NS2B. The five consecutive alanine residues are indicated in the bars for mutants. (**B**) The NS2B mutant of aa 115–119 loses interaction with HA-tagged KPNA6. HEK293T cells were co-transfected with HA-tagged KPNA6 and YFP-tagged NS2B mutants or EV, and the cell lysates were harvested at 48 hpt for co-IP with HA-tag antibody. Input lysates were included in the WB analysis. The relative co-precipitation efficiency of NS2B, shown below the IP images, was determined by normalizing the NS2B and KPNA6 levels in the IP to their input levels. (**C**) Schematic illustration of mutant NS2B with point mutations to alanine in the positions indicated. (**D**) Mutant NS2B-P115A and NS2B-G119A have much less presence than the mutant NS2B-F116A and WT NS2B in the KPNA6 precipitates. The relative co-precipitation efficiency of NS2B, shown below the IP images, was determined by normalizing the NS2B and KPNA6 levels in the IP to their input levels. (**E**) Mutant NS2B-P115A and NS2B-G119A induce little KPNA6 relocation in the co-transfected cells. Vero cells were co-transfected with plasmids of YFP-tagged KPNA6 and Myc-tagged NS2B or its mutants (P115A and G119A), or EV. The cells were fixed for IFA with an antibody against Myc tag 24 h post-transfection. Overlays of NS2B, KPNA6, and DAPI are shown. Pearson’s CC is indicated on the right. The bars in the lower right denote 10 µm.

To further determine the critical residues in NS2B required for interacting with KPNA6, we conducted alanine scanning mutagenesis on the 20 residues (aa 100–119) of NS2B by generating four alanine substitution mutants (Fig. 5A). Similarly, we co-transfected HEK293T cells with these mutants and HA-tagged KPNA6, followed by IP with the HA-tag antibody. The results indicate that all NS2B mutants except M4 were present in the KPNA6 precipitates (Fig. 5B). The M4 mutant contains three residue substitutions from residues PFAAG to AAAAA at the positions of aa 115–119 (Fig. 5A), suggesting that the three residues P115, F116, and G119 are potentially critical for the interaction. We then generated individual alanine substitutions for P115, F116, and G119 in NS2B (Fig. 5C). Co-transfection of HEK293T cells with each mutant NS2B and HA-tagged KPNA6, followed by IP for KPNA6, was performed. WB revealed that NS2B-P115A and NS2B-G119A mutants had much less co-precipitation with KPNA6 than the WT NS2B and NS2B-F116A (Fig. 5D), which suggests that both P115 and G119 residues appear to be critical for the NS2B interaction with KPNA6.

As NS2B was identified as the main viral protein inducing KPNA6 relocation, we next investigated whether these two residues are essential for NS2B to induce KPNA6 relocation. Vero cells were co-transfected with plasmids encoding the YFP-tagged KPNA6 and the Myc-tagged NS2B or its mutants NS2B-P115A and NS2B-G119A. Relocated KPNA6 was observed in a significantly greater number of cells co-transfected with wild-type NS2B than in the cells with either of the NS2B mutants (Fig. S2). Furthermore, in the cells with KPNA6 relocation, compared to wild-type NS2B, both NS2B mutants induced significantly less KPNA6 relocation, shown by a much lower Pearson’s CC (Fig. 5E). These results suggest both P115A and G119A are critical for NS2B to interact with KPNA6 and mediate its relocation.

### Substitution of either of the two NS2B residues in the ZIKV infectious cDNA clone hinders virus recovery

**Fig 6:**
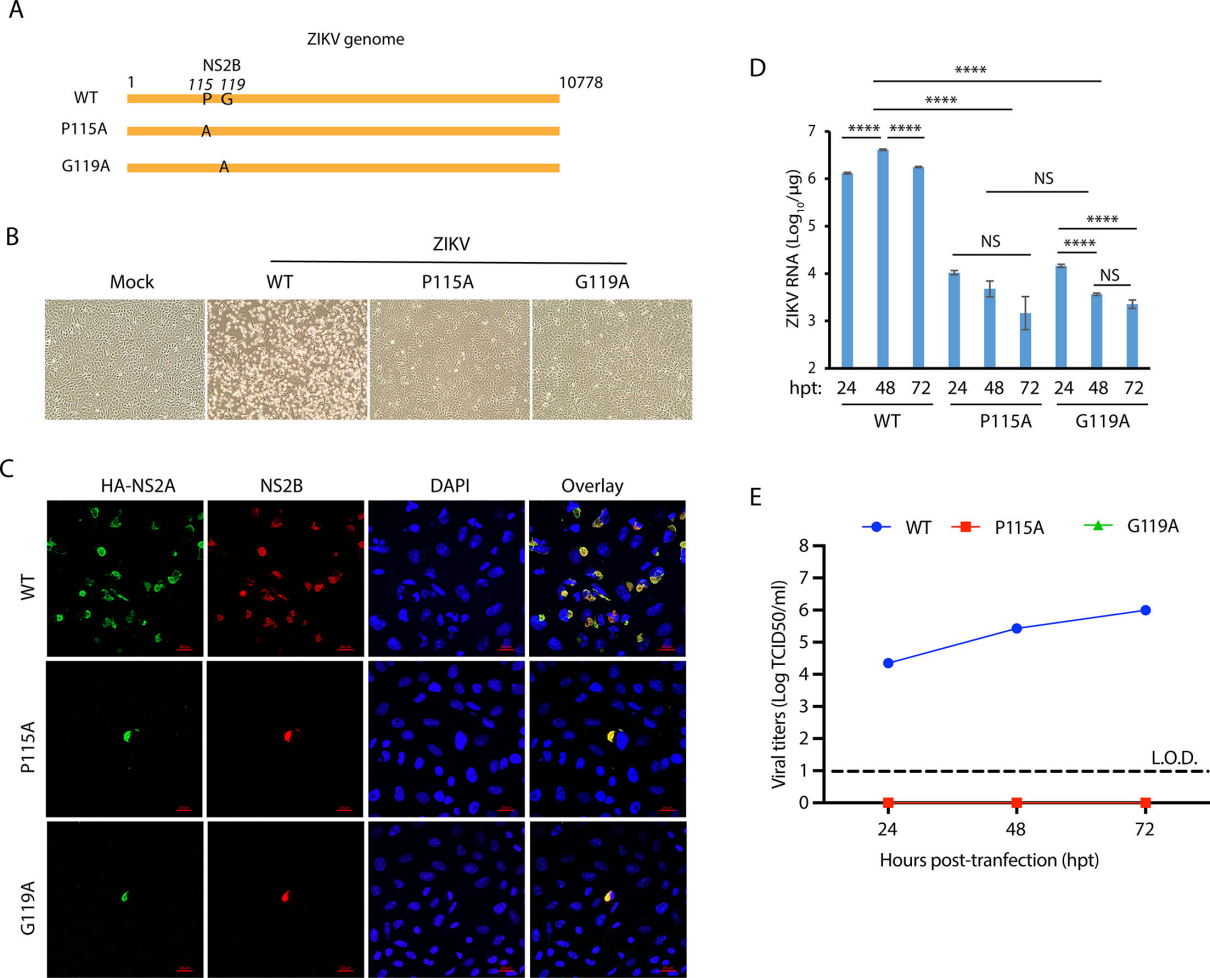
Mutations P115A and G119A in NS2B in the ZIKV infectious clone hinder virus recovery. (**A**) Schematic illustration of the residue substitutions in NS2B of the ZIKV cDNA clone. (**B**) No CPE is visible in the cells transfected with mutant ZIKV RNA, whereas transfection with the WT ZIKV RNA leads to severe CPE. Vero cells were electroporated with the RNA transcribed from ZIKV cDNA clones and observed daily. The images shown are from the cells 72 hpt. (**C**) IFA of cells transfected with ZIKV RNA 48 hpt. Vero cells were transfected with ZIKV RNA from pFLZIKV-HA-NS2A or its mutants, fixed 48 hpt, and probed with antibodies against HA-tag and NS2B. The scale bars in the lower right of the images denote 20 µm. (**D**) ZIKV RNA levels in the Vero cells at 24, 48, and 72 hpt. The ZIKV RNA copies in 1 µg total RNA are shown. NS, no significant difference; ****, *P* < 0.001. (**E**) Viral yield after transfection. The culture supernatant samples were collected daily and titrated. The means and standard errors (SD) from three independent samples are presented. Note that the error bars are smaller than the symbol size. L.O.D., the limit of detection.

To determine whether the two critical residues in the NS2B for KPNA6 interaction have any biological effect on ZIKV replication, we conducted site-directed mutagenesis of the ZIKV infectious cDNA clone pFLZIKV-HA-NS2A (28) and generated two mutants: pFLZIKV-HA-NS2A-NS2B-P115A and pFLZIKV-HA-NS2A-NS2B-G119A (Fig. 6A). These plasmids were used for *in vitro* transcription to generate full-length ZIKV RNA, followed by purification and transfection into Vero cells to recover the virus. The RNA from the wild-type cDNA clone induced severe cytopathic effect (CPE), whereas the two mutants showed no CPE at 72 hpt (Fig. 6B). To confirm ZIKV replication, we performed IFA with antibodies against the HA tag and ZIKV NS2B. The results showed clusters of HA-NS2A- or NS2B-positive cells in the well transfected with WT ZIKV RNA, whereas only sparse individual positive cells were visible in the wells transfected with the mutant ZIKV RNA (Fig. 6C). Total RNA was also isolated from the transfected cells to determine the ZIKV RNA levels with RT-qPCR. The results showed that ZIKV RNA levels in the mutants were significantly lower than in WT ZIKV by over 100-fold (Fig. 6D). The viral RNA levels peaked at 48 hpt for WT ZIKV, whereas they peaked at 24 hpt for the mutants. The viral titer of WT ZIKV increased over time, reaching 10^6^ TCID_50_/mL at 72 hpt, while there was no detectable mutant virus (Fig. 6E). Notably, both NS2B mutants displayed a similar pattern to the replication-defective control, an infectious clone carrying a mutation in the flavivirus-conserved NS5 polymerase active motif (GDD mutated to AAA) (Fig. S3). Since the cells transfected with mutant ZIKV RNA had no detectable live virus, blind passaging of the culture supernatant from the transfected cells of 72 hpt was done three times to amplify the virus. The virus from the WT clone caused severe CPE, whereas cells with the mutant clones showed no CPE at the third passage (data not shown). These results demonstrate that the mutations P115A and G119A in NS2B abolish virus replication, suggesting that these residues are essential for NS2B function and virus replication.

## DISCUSSION

Like most other (+)ssRNA viruses, ZIKV replicates in the cytoplasmic space. Our earlier study (13) and this work demonstrate that KPNA6 contributes to ZIKV replication. The interesting finding is that ZIKV infection induced KPNA6 relocation to the perinuclear region, where ZIKV replication occurs (26). The KPNA6 relocation was confirmed with cytoplasmic and nuclear fractionation of ZIKV-infected cells. As ZIKV infection progresses, an increasing amount of KPNA6 relocates to the cytoplasm. IFA of infected cells at multiple time points after inoculation further verified the translocation of KPNA6, as Pearson’s CC with DAPI staining decreased with increasing ZIKV RNA levels. These results demonstrate that ZIKV infection induces KPNA6 accumulation in the perinuclear region, providing a clue to KPNA6’s contribution to virus replication.

The KPNA6 accumulation in the perinuclear region in ZIKV-infected cells prompted us to identify the viral protein responsible for this effect. Viral protein screening showed that NS2B induced the greatest KPNA6 relocation when co-expressed with KPNA6. NS2B is a cofactor of the NS2B-NS3 protease, and the central hydrophilic loop alone is sufficient for the protease activity (22, 23, 29). The transmembrane helices at both N- and C-termini of NS2B are anchored in the ER, with the hydrophilic loop on the cytoplasmic side (26, 27). The transmembrane domains of NS2B of the Japanese encephalitis virus (JEV), another flavivirus, have been shown to contribute to viral RNA synthesis and virion assembly (30). Systemic mutagenesis of flavivirus-conserved residues in the transmembrane domains of JEV NS2B showed that mutations G37L and P112A resulted in lower RNA synthesis and virion assembly without affecting NS2B-NS3 protease activity. Further study showed that the P112A mutation affected NS2A-NS2B interaction (30). Among the NSPs of West Nile virus (WNV), NS2B interacts with NS2A, NS3, NS4A, and NS4B (31).

KPNA6 recognizes and binds its cargo through NLS on the cargo protein (32). However, no predicted NLS was observed in NS2B, prompting us to define the NS2B-binding site in KPNA6. The KPNA6 deletion analysis showed that the N-terminal half interacted with NS2B. To exclude the involvement of the IBB domain in the interaction, we tested the IBB-deleted KPNA6 and found that it interacted with NS2B, as did the full-length protein. This result led us to speculate that the major groove formed by ARM2–4 domains was the possible NS2B-binding site. Mutations of the W and N residues that are highly conserved and are known to contribute to cargo binding in ARMs 2 and 3 of the major groove resulted in much less NS2B in the KPNA6 precipitates. The results indicate that the major groove is the binding site for NS2B, and that all the W and N residues tested in the ARM2–3 domains contribute to the pocket that forms for NS2B binding. This is consistent with the NLS binding pattern of KPNA in the major or minor groove (21).

Our data demonstrate that the two residues at the C-terminal helix of NS2B are required for interaction with KPNA6. Since NS2B lacks a predicted NLS, we began with a structural analysis to identify the domain that could interact with KPNA6. We noted that the C-terminal helix was potentially the binding site. NS2B deletion mutants in the region were constructed and used to verify the binding. As a result, 20 residues (aa 101–120) were found to be needed in the interaction with KPNA6. These residues are located in the C-terminal two α-helixes (94–109 and 113–128) (25). Deleting the first or the second 10 residues in the stretch caused a significant decrease in the interaction, indicating that such deletions might interrupt the helix formation needed for KPNA6 binding. Next, we performed alanine-scanning mutagenesis to identify the specific residues essential for the KPNA6-NS2B interaction and showed that residues 115–119 were required for KPNA6 binding. The protein level of NS2B-M4 was much lower than that of other deletion mutants shown in the input (Fig. 5B), suggesting that mutation of the three residues also affects the protein expression. Multiple attempts failed to shore up the expression. However, this low expression should not account for the absence of NS2B-M4 in the KPNA6 precipitates, as extended image scanning was unable to pick up any signal for the M4 in the IP image. Single residue substitutions were then conducted to determine the two residues, P115 and G119, in the second α-helix (113–128) of C-terminal NS2B, which were identified as needed for the interaction. Previous nuclear magnetic resonance-based structural analyses have characterized the C-terminal helices of flavivirus NS2B as membrane-associated (33, 34). Accordingly, P115 and G119 are likely positioned within the C-terminal membrane-associated helix. Proline and glycine residues are known to influence helix geometry and flexibility in transmembrane domains (35). Substitution of these two residues with alanine may therefore alter the structure of the helix, leading to some change in NS2B and affecting its interaction with KPNA6. Thus, the effects of the mutations may reflect subtle changes in NS2B structure rather than loss of secondary structure. The two residues, P115 and G119, are in the C-terminal hydrophobic helix of NS2B and are dispensable for the NS2B/NS3 protease activity. Yet, substituting either P115 or G119 with alanine hinders ZIKV replication. These results suggest that non-protease cofactor functions of NS2B, such as interaction with KPNA6, are affected by the mutations and are needed for viral replication. Because NS2B anchoring in the ER contributes to the formation of viral replication factories (10), mutations in the two residues might affect this essential step in the virus life cycle. Although the precise structural mechanism underlying the interaction with KPNA6 remains to be defined, our data support a role for P115 and G119 in NS2B activities beyond its protease cofactor function. Further work is needed to elucidate the exact roles of the two residues in NS2B function and to determine whether KPNA6 facilitates ZIKV replication complex formation or other aspects that are essential for viral replication.

The role of NS2B beyond its function as a cofactor of the NS2B-NS3 protease is less understood. It is thought that the transmembrane domains of NS2B of JEV contribute to viral RNA replication and virion assembly (30). The NS2B of JEV was also found to interact with host factor SPCS1 (Signal Peptidase Complex Subunit 1), and both N- and C-terminal transmembrane domains of NS2B interact with SPCS1 (36). SPCS1 is needed for proper cleavage of prM and E proteins, and the loss of SPCS1 reduces virus yield of the Flaviviridae family members: WNV, dengue virus (DENV), ZIKV, yellow fever virus, JEV, and hepatitis C viruses (37). In DENV, the transmembrane helix is implicated in oligomerization and association with host membranes, inducing destabilization by pore formation (38). NS2B of DENV also inhibits type-I interferon production by inducing the degradation of cyclic GMP-AMP synthase, a cytosolic DNA sensor (39). Our previous data show that ZIKV infection induces an elevation in KPNA6 levels and that depleting KPNA6 significantly reduces ZIKV replication (13). This study indicates that ZIKV induces the relocation of KPNA6 to the perinuclear region and that NS2B interacts with KPNA6, likely via the C-terminal transmembrane domain. This interaction may contribute to NS2B’s non-cofactor roles. Further study is needed to determine its exact contribution to the virus life cycle.

Many viruses, especially those with DNA genomes, are known to exploit KPNAs for their benefit. DNA viruses generally deliver their genetic elements into the nucleus for propagation or integration into the host chromosome for latent infection. Importins like KPNA6 play essential roles in this process and are thus pivotal host-dependency factors. For example, parvovirus delivers its DNA into the nucleus through importin α and β (40). Antibodies against KPNA2 and KPNB1 inhibit human papillomavirus replication. Besides, herpesvirus and adenovirus rely on importins for their nuclear entry (41). Previous studies on virus modulation of KPNAs focused on interference with host cell signaling. For example, Ebola virus VP24 binds KPNA1 and blocks STAT1 nuclear translocation (42, 43); the ORF6 product of severe acute respiratory syndrome coronavirus disrupts nuclear import of STAT1 by tethering KPNA2 to the ER/Golgi membrane (44); PRRSV nsp1β protein blocks STAT1 nuclear entry by inducing degradation of KPNA1 (45, 46).

Among ZIKV proteins, NS3 and NS5 are known to translocate to the nucleus in infected cells by KPNA1 and KPNA2, respectively (47, 48). The ZIKV C protein is also translocated to the nucleus by importin-7 (IPO7), a member of the importin-beta family (49). However, the C of West Nile virus, another flavivirus, relocates to the nucleus by KPNA4 (50). The nuclear translocation of ZIKV C and NS5 proteins was unaffected by KPNA6 deletion (data not shown). Thus, the nuclear translocation of these ZIKV proteins does not appear to require KPNA6 or to involve the viral genome synthesis directly.

The KPNA6 has been found to assist the nuclear transport of viral RNA polymerase of the mammalian influenza virus, while avian viruses use KPNA4, showing the cell tropism and host adaptation of the viruses (51). Another study shows that KPNA2 and KPNA6 are positive regulators for human-like but not avian-like influenza virus polymerase activity, in addition to nuclear transport (52). It was also shown that mice lacking KPNA6 were less susceptible to human-like influenza virus. KPNA6 is required for the influenza A virus replication in the alveolar epithelium, leading to severe lung damage and death of mice, whereas KPNA6-deficient mice have restricted virus replication to the bronchial epithelium with enhanced survival (53). The KPNA6 supports influenza virus polymerase by interacting with the viral ribonucleoprotein complex and ANP32, a host factor essential for the polymerase activity (54). These data suggest that KPNA proteins, especially KPNA6, can act as proviral factors for the influenza A virus by facilitating nuclear transport and viral polymerase activity.

As an obligatory intracellular agent, ZIKV recruits host factors and modulates others to generate a favorable milieu for replication. ZIKV upregulates KPNA6 (13) and downregulates KPNA2 (55). These findings show that ZIKV perturbs the transport factors to its advantage. In conclusion, ZIKV infection induces the relocation of KPNA6 to the perinuclear region, potentially via NS2B, to facilitate efficient viral replication. The molecular basis of the NS2B interaction with KPNA6 is the two residues in the C-terminal helix. These two residues in NS2B appear to be critical for ZIKV replication, as replacing either of them inhibits viral replication. These findings offer valuable insights into ZIKV-host interactions and could facilitate the development of novel antiviral strategies.

## MATERIALS AND METHODS

### Cells, viruses, and chemicals

HEK293T (ATCC CRL-3216) and Vero (ATCC CCL81) cells were maintained in Dulbecco’s modified Eagle medium supplemented with 10% fetal bovine serum. ZIKV strain PRVABC59 (ATCC VR-1843) was used in this study. Vero cells were inoculated at the MOI described in the figure legends or results. Virus yields were titrated in Vero cells by 10-fold serial dilutions for the median tissue culture infectious dose (TCID_50_) as log_10_/mL (55). For transient expression, plasmid DNA was transfected into cells with jetOPTIMUS DNA transfection Reagent (Polyplus transfection, New York, NY) according to the manufacturer’s instructions.

For cytoplasmic and nuclear fractionation, NE-PER Nuclear and Cytoplasmic Extraction Reagents (Thermo Fisher Scientific, Waltham, MA) were used according to the manufacturer’s instructions.

### Plasmid and site-directed mutagenesis

Full-length KPNA6 (GenBank accession number NM_012316) and KPNA6 deletion mutants were cloned into the pCAGEN vector with a Myc tag or HA tag at the N-terminus as previously reported (13). The YFP-tagged KPNA6 was constructed by cloning full-length KPNA6 into the pCDNA3-VenusC1 vector in this study. Cloning full-length NS2B and other viral proteins of ZIKV PRVABC59 was described previously (55). The C, prM, E, NS1, NS2A, NS2B, NS3, NS4A, NS4B, and NS5 of ZIKV were also cloned into the pCAGEN vector with a Myc tag in this study. NS2B deletion mutants, KPNA6 mutants, or NS2B mutants with amino acid substitutions were done with the Q5 Site-Directed Mutagenesis Kit (New England Biolabs, Ipswich, MA) according to the manufacturer’s instructions. All primers used for the plasmid construction are listed (Table 1). All in-house-constructed plasmids were sequenced by Plasmidsaurus using Oxford Nanopore Technology, with custom analysis and annotation to verify the inserts.

**TABLE 1:** Primers used in this study

Primer^*a*^	Sequence (5’ to 3’)^*b*^	Target gene/use
PR-NS2B-F5	CTCGAGCATGCATCTAGAGGGCCCTATTC	NS2B/D3
PR-NS2B-R5	CGCTCCAGCTGCAAAGGGTATGGCTATTG	NS2B/D3
PR-NS2B-F6	TGGTACGTATACGTGAAGACTGGAAAAAGG	NS2B/D2
PR-NS2B-R6	GTTCATGCCACAGATGGTCATCAGGACCAC	NS2B/D2
PR-NS2B-F7	CCAATAGCCATACCCTTTGCAGCTGGAGCG	NS2B/D1
PR-NS2B-R7	CTTGAGTATGATCTCTCTCATGGGGGGACC	NS2B/D1
NS2B-m100-104-F1	CGCAGCTACCATCTGTGGCATGAAC	NS2B/M1
NS2B-m100-104-R1	GCCGCCGCGAGTATGATCTCTCTCATGG	NS2B/M1
NS2B-m105-109-F1	CGCAGCTAACCCAATAGCCATACCC	NS2B/M2
NS2B-m105-109-R1	GCCGCCGCCATCAGGACCACCTTGAG	NS2B/M2
NS2B-m110-104-F1	CGCAGCTCCCTTTGCAGCTGGAGCG	NS2B/M3
NS2B-m110-104-R1	GCCGCCGCCATGCCACAGATGGTCATCAG	NS2B/M3
NS2B-m115-119-F1	CGCAGCTGCGTGGTACGTATACGTG	NS2B/M4
NS2B-m115-119-R1	GCCGCCGCTATGGCTATTGGGTTCATG	NS2B/M4
NS2B-P115A-F	AATAGCCATAGCGTTTGCAGCTG	NS2B/P115A
NS2B-P115A-R	GGGTTCATGCCACAG	NS2B/P115A
NS2B-F116A-F	AGCCATACCCGCGGCAGCTGGAG	NS2B/F116A
NS2B-F116A-R	ATTGGGTTCATGCCAC	NS2B/F116A
NS2B-G119A-F	CTTTGCAGCTGCGGCGTGGTACG	NS2B/G119A
NS2B-G119A-R	GGTATGGCTATTGGGTTC	NS2B/G119A
KPNA6-F1	CC*GAATTC*GAGACCATGGCGAGCCCAGGG	KPNA6/WT
KPNA6-R1	GC*CTCGAG*TTATAGCTGGAAGCCCTCC	KPNA6/WT
KPNA6-R4	G*CTCGAG*TCATAGGCGAGACAGTACAGGCAAAC	KPNA6/D1
KPNA6-F5	A*GAATTC*CCTGTACTGTCTCGCCTACTC	KPNA6/D3
KPNA6-R5	G*CTCGAG*TCAATAAGGATTGACCCCTGAGCC	KPNA6/D2
ARM2-W147A+N151A-F	TAACGGCGATTGCCTCTGGAACCTCTCAGCAG	KPNA6/M1
ARM2-W147A+N151A-R	GAGCCGCGGCAGCTTCAAACTGTAATGTACAATTC	KPNA6/M1
ARM3-W189A+N193A-F	GGAGCGATAGCTGGAGATAGCTCTGTTTGCC	KPNA6/M2
ARM3-W189A+N193A-R	CAGTGCCGCGACTGCCTGTTCCTGAACATCCTC	KPNA6/M2
ARM4-W232A+N236A-F	GTCAGCGCTCTGCCGAGGGAAAAACCCACCC	KPNA6/M3
ARM4-W232A+N236A-R	AGGGCCGCGACTGCATTCCGTGTCATCGTC	KPNA6/M3
NS5-GDD-F2	CTGCTTGCGTTGTGAAACCAATTG	NS5/ΔGDD
NS5-GDD-R2	CTGCACTGACTGCCATTCGTTTGAGC	NS5/ΔGDD

^*a*^ F, forward primer; R, reverse primer.

^*b*^ The italicized letters indicate restriction enzyme cleavage sites for cloning.

Plasmid pFLZIKV-HA-NS2A (a gift from Dr. Pei-Yong Shi from the University of Texas Medical Branch) is a ZIKV infectious cDNA clone containing HA-tagged NS2A with full-length cDNA of ZIKV strain FSS13025 (28). The mutant ZIKV cDNA clone was constructed with P115A or G119A of NS2B, or NS5ΔGDD, using the Q5 Site-Directed Mutagenesis Kit. The whole plasmid sequencing was performed by Plasmidsaurus using Oxford Nanopore Technology with custom analysis and annotation. *In vitro* RNA transcription was done with HiScribe T7 ARCA mRNA Kit (New England Biolabs) on the pFLZIKV-HA-NS2A and P115A or G119A mutants. The 4 μg purified mRNA was electroporated into Vero cells (1.6 × 10^6^ cells) with a Neon Electroporation System (Thermo Fisher Scientific) at the following conditions: 1,400 volts, 30 ms, and 1 pulse. After electroporation, 1 × 10^5^ cells/well were seeded into a 24-well plate with/without coverslips containing culture media and incubated at 37°C with 5% CO_2_. At given time points post-transfection, cell culture supernatant was collected for titration; cell lysate was harvested for RNA extraction; or cells grown on a coverslip were fixed for immunofluorescence assay as described. The cell culture supernatant was collected at 72 h post-transfection and used to inoculate fresh Vero cells for further propagation. The virus yield of the third passage of WT ZIKV was titrated.

### IFA

IFA was performed as previously reported (13). Briefly, paraformaldehyde-fixed cells were probed with primary antibodies against KPNA6 (Proteintech Group, Inc., Rosemont, IL), ZIKV NS2B (GeneTex, Inc., Irvine, CA), HA tag (Thermo Fisher Scientific), Myc tag (ABclonal, Woburn, MA), and dsRNA (J2) (Jena Bioscience, Germany). Fluorescein-conjugated secondary antibodies used include goat anti-rabbit IgG (H&L) DyLight 549 and goat anti-mouse IgG (H&L) DyLight 488 (Rockland Immunologicals, Inc., Limerick, PA). SlowFade Gold antifade reagent containing DAPI (4′,6′-diamidino-2-phenylindole) (Thermo Fisher Scientific) was used for coverslip mounting. Imaging was done with a Zeiss LSM800 confocal microscope and Zen software, which was also used to evaluate the degree of co-localization of two fluorophores as shown by Pearson’s CC: a value of 1 indicates perfect correlation, 0 indicates no correlation, and −1 indicates ideal anti-correlation.

### WB

Cell lysate or other samples in Laemmli sample buffer were subjected to SDS-PAGE and WB as described previously (13, 55). Primary antibodies against PARP1 (Santa Cruz Biotechnology, Inc., Dallas, TX), KPNA6 (GeneTex), GAPDH (Santa Cruz), GFP (BioLegend, San Diego, CA), HA tag (Thermo Fisher Scientific), Myc tag (ABclonal), β-actin (ABclonal), and tubulin (Sigma-Aldrich, St. Louis, MO), as well as antibodies against ZIKV NS2B (GeneTex) and ZIKV E (GeneTex), were used in this study. The secondary antibodies conjugated with horseradish peroxidase included goat anti-mouse or anti-rabbit IgG (Rockland). A chemiluminescence substrate was used to reveal the specific reactions, which were acquired in the linear range of digital intensity without saturated pixels. Densitometric analysis of the WB images was done with the QuantityOne Program, version 4.6 (Bio-Rad Laboratories, Hercules, CA).

### IP

Cells were lysed with RIPA buffer (Thermo Fisher Scientific) supplemented with a protease inhibitor cocktail (Sigma) at given time points. Clarified cell lysates by centrifugation were incubated with specific antibodies indicated in the figures, followed by capturing with protein A/G magnetic beads (Bimake.com, Houston, TX) as described previously (13). The IP eluate was subjected to WB to detect target proteins in the precipitate.

### Computational analysis of protein interaction

The secondary structural analysis of ZIKV NS2B was done with DNASTAR Lasergene version 17 (DNASTAR, Inc., Madison, WI). The transmembrane domain prediction of NS2B was performed by the DeepTMHMM analysis (https://dtu.biolib.com/DeepTMHMM). The AlphaFold2 web server was used to predict the 3D structure of NS2B (56). The 3D structure of KPNA6 (4UAD) was downloaded from the Protein Data Bank (https://www.rcsb.org/structure/4UAD). Docking of NS2B and KPNA6 was conducted with the ZDOCK server (https://zdock.wenglab.org/) and HADDOCK web server (https://rascar.science.uu.nl/haddock2.4/). Molecular graphics and analyses were performed with UCSF ChimeraX (57).

### RNA isolation and real-time PCR

Total RNA was isolated from Vero cells with the PureLink RNA Mini Kit (Thermo Fisher Scientific) following the manufacturer’s instructions. Reverse transcription and real-time PCR (RT-qPCR) were performed as described previously (13, 55). In addition, a standard curve was generated using plasmids encoding ZIKV NS5 (pCDNA3-VenusC1-HL-NS5) (55) to quantitatively estimate the viral RNA copy number. The real-time PCR primers used for ZIKV were described previously (55). All experiments were repeated at least three times, with each conducted in triplicate.

### Statistical analysis

Differences between the two groups were assessed using Student’s *t*-test. A two-tailed *P*-value of 0.05 was considered significant.

## Data Availability

Data are presented in this article and its supplemental material.

## SUPPLEMENTAL MATERIAL

Supplemental material jvi.02009-25-s0001.pdf Fig. S1 to S3.

ASM does not own the copyrights to Supplemental Material that may be linked to, or accessed through, an article. The authors have granted ASM a non-exclusive, world-wide license to publish the Supplemental Material files. Please contact the corresponding author directly for reuse.
