# Supplementary material for: Zika virus recruits karyopherin α6 for efficient replication via NS2B
Source: J Virol. 2026 May 20;100(6):e02009-25. doi: 10.1128/jvi.02009-25 (PMC13288816; doi:10.1128/jvi.02009-25)
Supplement: Supplemental material — Fig. S1 to S3. [file jvi.02009-25-s0001.pdf]

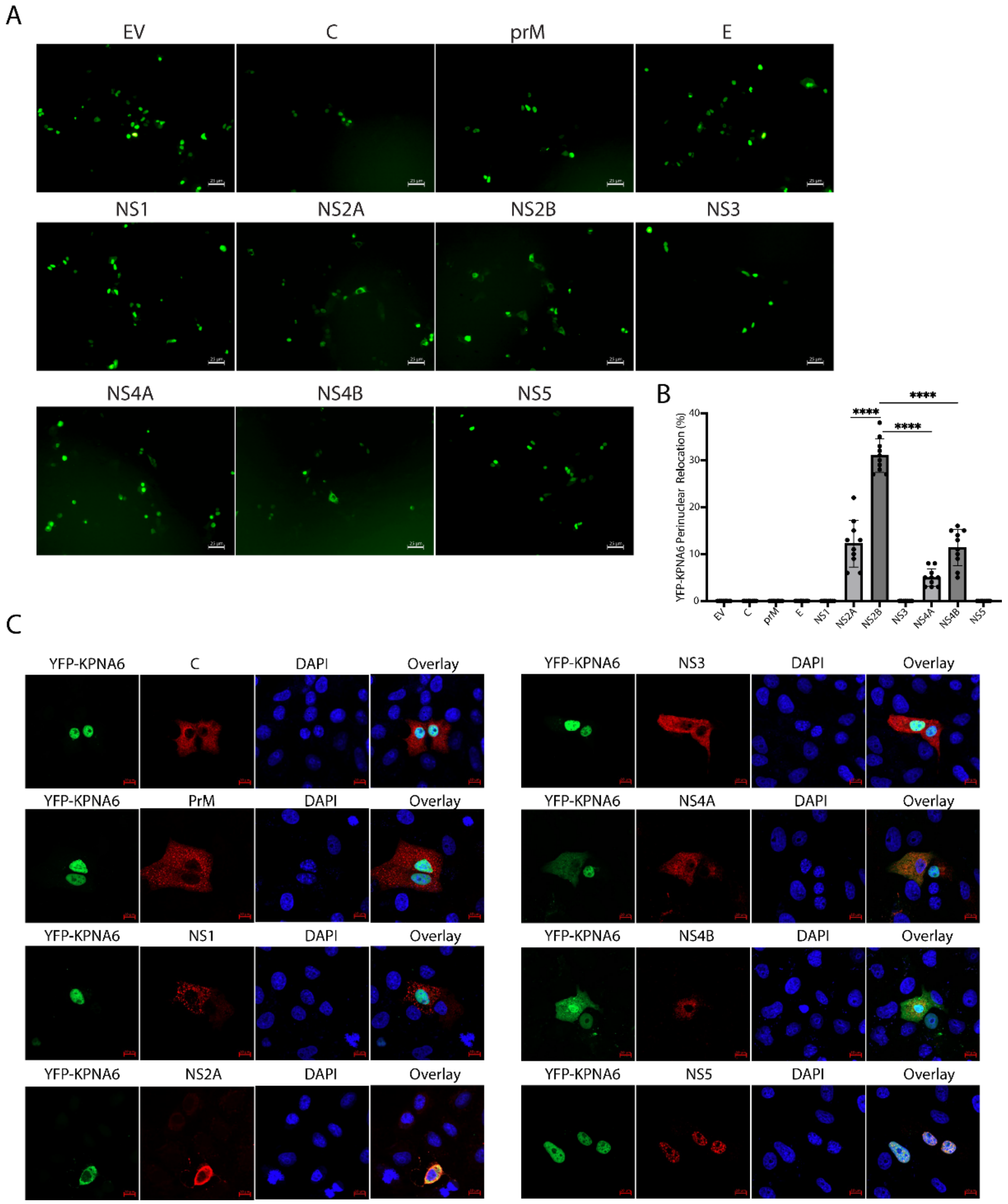

Supplemental Figure 1: Screening of ZIKV viral proteins for KPNA6 relocation. A. Representative fluorescence images of Vero cells co-transfected with plasmids of YFP-tagged KPNA6 and individual Myc-tagged ZIKV viral proteins (C, prM, E, NS1, NS2A, NS2B, NS3, NS4A, NS4B, and NS5) or empty vector (EV). The cells with green fluorescence, which indicate KPNA6, were observed and captured under a fluorescence microscope (Olympus CKX53) 24 hours post-transfection. The scale bars in the lower right of each image denote 25 µm. B. The percentages of cells with YFP-KPNA6 relocated from the nucleus to the perinuclear region. For each image (field), the total number of YFP-positive cells was counted, and those with perinuclear YFP-tagged KPNA6 were counted to calculate the relocation percentage. Each dot above and in the bars represents one observation field (n = 10 fields, with ≥ 200 YFP-positive cells counted per sample). The KPNA6 relocation percentages are presented as means with standard errors. Data shown are representative of at least three independent experiments. ****, P < 0.001. C. Presence of YFP-tagged KPNA6 and individual Myc-tagged ZIKV proteins (C, prM, NS1, NS2A, NS3, NS4A, NS4B, and NS5) in co-transfected cells. Vero cells were co-transfected with the plasmids encoding YFP-tagged KPNA6 and Myc-tagged ZIKV proteins. The cells were fixed for IFA with an antibody against the Myc-tag 24 h post-transfection. The overlays of KPNA6, individual ZIKV proteins, and DAPI are shown. The scale bars in the lower right of each image denote 10 µm.

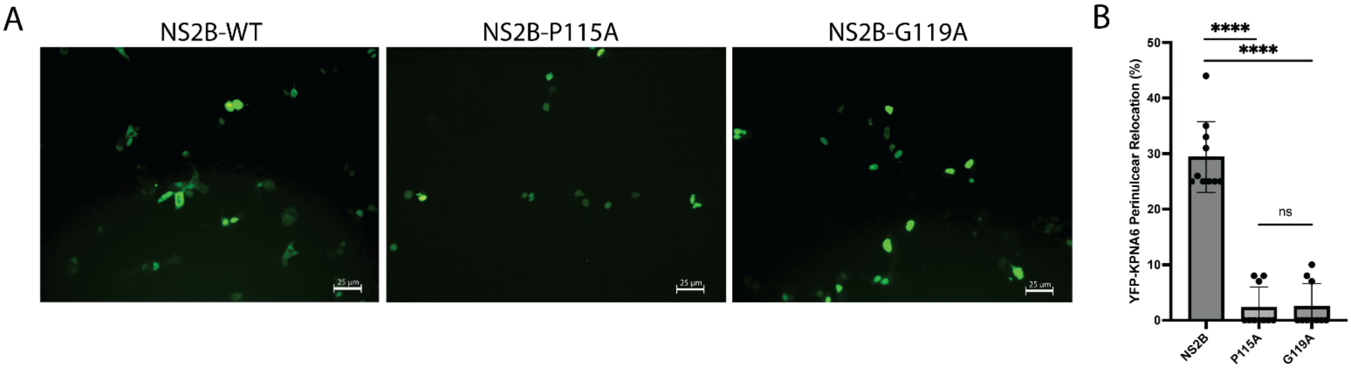

Supplementary Figure 2: NS2B mutants induce less KPNA6 relocation. Vero cells were co-transfected with plasmids encoding the YFP-tagged KPNA6 and the Myc-tagged NS2B or its mutants NS2B-P115A, NS2B-G119A. Cells exhibiting green fluorescence, indicative of KPNA6, were observed and captured under a fluorescence microscope (Olympus CKX53) 24 hours post-transfection. The scale bars in the lower right of each image denote 25 µm. B. The percentages of cells with YFP-KPNA6 relocated from the nucleus to the perinuclear region. For each image (field), the total number of YFP-positive cells was counted, and those with perinuclear YFP-tagged KPNA6 were counted to calculate the relocation percentage. Each dot above and in the bars represents one observation field ($n = 10$ fields, with ≥ 200 YFP-positive cells counted per sample). The KPNA6 relocation percentages are presented as means with standard errors. Data shown are representative of at least three independent experiments. ****, $P < 0.001$. ns, no significant difference.

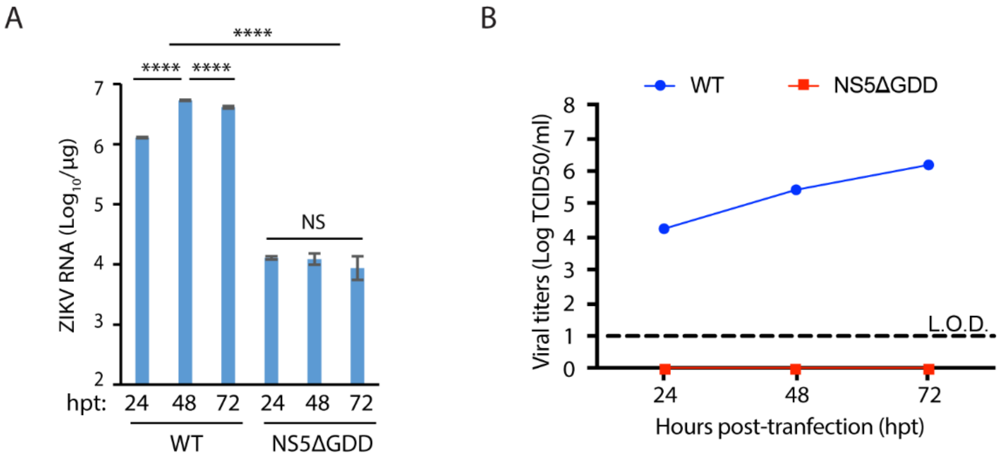

Supplementary Figure 3: Comparison of wild-type and replication-defective mutant infectious clone. A. ZIKV RNA levels in the Vero cells at 24, 48, and 72 hpt. Vero cells were electroporated with RNA transcribed from ZIKV cDNA clone, either pFLZIKV-HA-NS2A or its NS5ΔGDD mutant. The cells were harvested at 24, 48, and 72 hpt for RNA isolation and RT-qPCR. The ZIKV RNA copies in 1 µg total RNA are shown. NS, no significant difference; ****, $P < 0.001$. B. Viral yield after transfection. The culture supernatant samples were collected daily and titrated. The means and standard errors (SD) from three independent samples are presented. Note that the error bars are smaller than the symbol size. L.O.D.: the limit of detection.
